# Population-level bistability in *Pseudomonas aeruginosa* quorum sensing

**DOI:** 10.1128/mbio.01713-25

**Published:** 2025-09-10

**Authors:** Bryce M. Pettit Estell, Martin Schuster

**Affiliations:** 1 Department of Microbiology, Oregon State University 2694, Corvallis, Oregon, USA; Indiana University Bloomington, Bloomington, Indiana, USA

**Keywords:** quorum sensing, bistability, hysteresis, steady state, *Pseudomonas aeruginosa*

## Abstract

Quorum sensing (QS) is a widespread signaling mechanism in bacteria that coordinates collective behaviors according to population density. A foundational assumption in this field is that QS functions as a gene expression switch that synchronizes responses at the population level. While some studies indeed report homogeneous on/off transitions, others report heterogeneity at the cellular level, challenging the canonical view. To provide a formal context for these observations, we examined QS behavior at physiological steady state in the model bacterium *Pseudomonas aeruginosa*. We measured the expression of a central *las*-system component, the positively autoregulated signal synthase gene *lasI*, at the population and single-cell level. In support of the canonical view and predictions from mathematical modeling, we show that the *las* system exhibits population-level bistability, with the entire unimodal population of cells switching synchronously between two stable states. We also show that bistable state switching exhibits hysteresis, indicative of memory within the system, with induced cells maintaining activation at considerably lower densities than previously uninduced cells. We confirm these behaviors in a subset of other *las*-QS controlled genes. Our study experimentally proves population-level bistability as a central, emergent property in a native QS system, with implications for physiology, pathogenesis, and synthetic biology.

**IMPORTANCE:**

This research investigates quorum sensing (QS), a common bacterial communication mechanism that controls processes like virulence, biofilm formation, and microbial warfare. Our study experimentally proves the long-standing notion that native QS can function as a true genetic switch that synchronizes all-or-none responses in a population of cells. We employ the well-understood LasI/LasR QS system of the opportunistic pathogen *Pseudomonas aeruginosa* as a model. We show that the switch is bistable, with stable on and off states, and that it is hysteretic, with a type of memory that makes state-switching dependent on the initial condition. These properties impart robustness and stability to environmental changes akin to cellular developmental pathways; they have general implications for infection and its control, as well as genetic circuit design.

## INTRODUCTION

Quorum sensing (QS) is a common mechanism of cell-cell communication in bacteria (1–3). It controls a range of different behaviors that are often cooperative in nature, such as collective nutrient acquisition, biofilm formation, dispersal, or microbial warfare. These behaviors generally involve shared, secreted products referred to as public goods (4, 5). QS is defined as the production and response to diffusible signaling molecules termed autoinducers (AIs) that enable the regulation of gene expression according to population cell density (1, 6).

**Fig 1:**
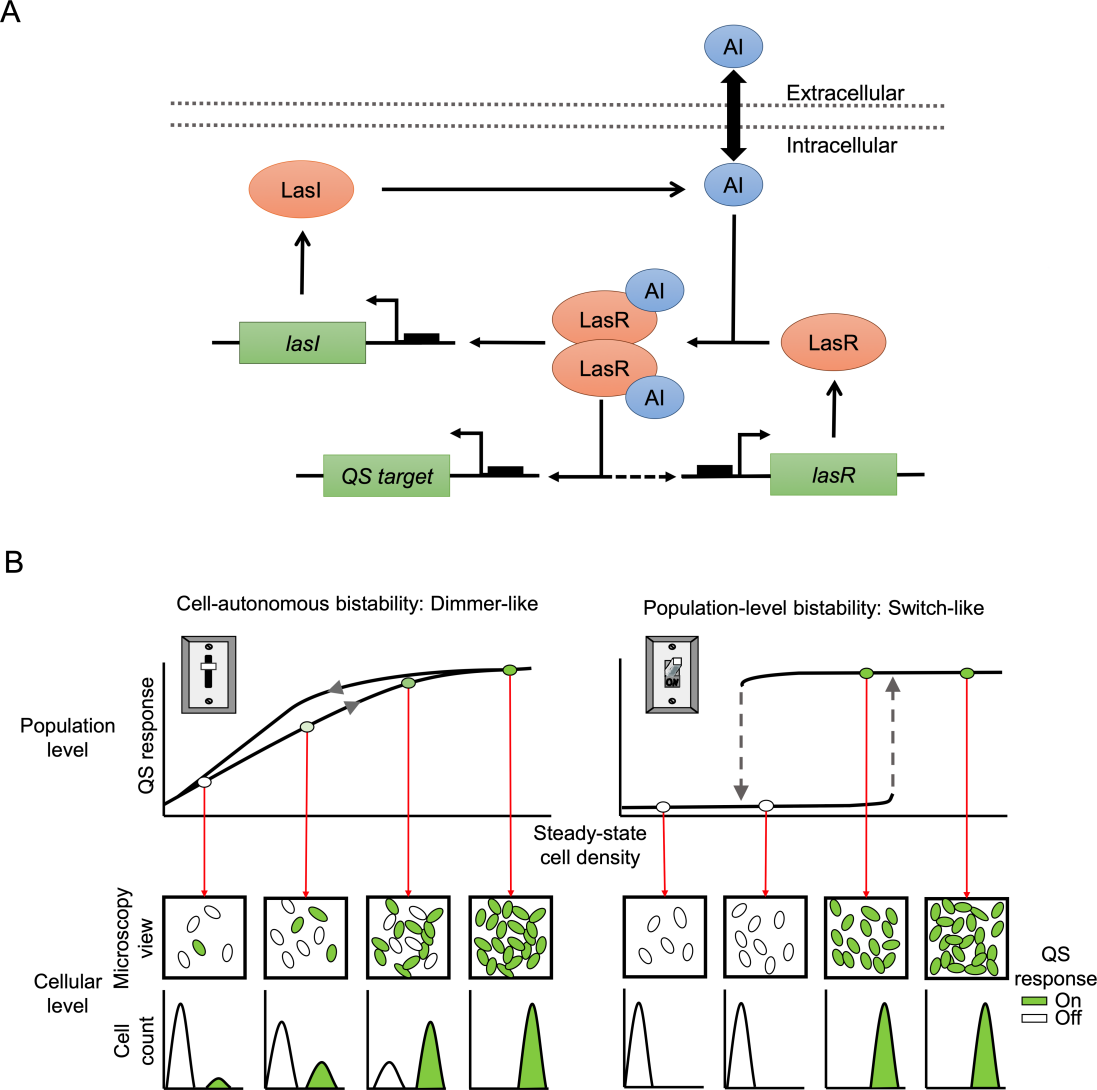
QS circuit and emerging response types. (**A**) Depiction of the LasR/LasI (*las*) QS circuit in *P. aeruginosa*. Genes are shown in green, proteins are shown in red, and the small molecule autoinducer (AI) 3OC12-HSL is shown in blue. 3OC12-HSL moves in and out of the cell. Inside the cell, the signal receptor LasR combines with AI to form a signal-bound dimer. This complex binds to conserved promoter sequences (black boxes) to activate the transcription of target genes. These genes include *lasI* and, to a lesser extent, *lasR* itself (dashed arrow), causing a positive feedback loop in the QS circuit. (**B**) Possible steady-state QS response types emerging from cellular QS networks on the population and individual cell level. Left, cell-autonomous bistability, representing a dimmer-like response with a gradual increase in population-level expression. The response curves are offset, depending on the direction of change (gray arrowheads). Right, population-level bistability, representing a switch-like response with two disjointed branches and distinct transition thresholds depending on the direction of change (gray dashed arrows). Cellular-level responses are shown below each population-level response. The microscopy view distinguishes QS-off cells (white ovals) and QS-on cells (green ovals). The histogram format at the bottom shows the relative abundance of on and off subpopulations, as would be obtained from single-cell flow cytometry. For cell-autonomous bistability, only one set of cellular responses is shown, for clarity.

Among the best-understood QS systems is the acyl-homoserine lactone (AHL) signaling circuit in the opportunistic pathogen *Pseudomonas aeruginosa* (7, 8). In this organism, AHL-QS controls the expression of hundreds of genes, including extracellular enzymes, toxins, metabolites, and secretion machinery (9, 10). Our particular focus is on the LasI/LasR (*las*) system in *P. aeruginosa* (Fig. 1A). This system consists of the AHL synthase LasI and the AHL receptor and transcriptional activator LasR. LasI generates 3-oxo-dodecanoyl-homoserine lactone (3OC12-HSL) that accumulates during culture growth and binds to LasR (11). LasR-3OC12-HSL functions as a dimer that binds to conserved sequence elements in target promoters to activate gene expression (12). Signal-bound LasR also activates *lasI*, and to a lesser extent, *lasR* itself, resulting in a positive feedback loop (13, 14).

Positive feedback and receptor dimerization are two key QS network elements that enable bistability (15–17). Bistability is an emergent property of a dynamical system in which its long-term behavior, assessed under constant environmental conditions, is characterized by two disjointed branches of stable steady states (18). This case represents a true genetic switch, in which the cellular QS system is either in the uninduced “off” state or the fully induced “on” state (Fig. 1B). Bistability is accompanied by hysteresis, in which state-switching depends on the history of the system, such that the switch from off to on occurs at a higher cell density than the switch from on to off (19).

Intriguingly, a bistable response determined by the specific network architecture at the cellular level can either produce a bistable or a graded response at the population level (Fig. 1B). Modeling data show that the response type depends on the strength of extracellular signal feedback between cells (20). A bistable all-or-none response, in which all cells switch between states in a concerted fashion, is possible if QS induction is mediated by the extracellular accumulation of signal. This response type is referred to as population-level bistability and has been considered a hallmark of canonical QS (6, 15, 20). In contrast, a graded population response is achieved when feedback is largely from the noise-triggered intracellular accumulation of signal (20). Potential benefits of these different response types could be robustness to stimulus variations in the case of population-level bistability, adjustment to intermediate stimuli and bet-hedging in the case of cell-autonomous bistability, and the optimization of QS-controlled behaviors with different properties in both cases (18, 21–23).

Steady-state behaviors such as bistability define the boundaries between which microbial responses to environmental changes unfold (24). However, only two studies have directly examined QS response types at physiological steady state, with discrepant findings (16, 19). They both utilized AHL-QS components (*luxI* and *luxR*) of the marine bacterium *Vibrio fischeri*, albeit expressed in the heterologous host *Escherichia coli*. One study suggests that dual positive feedback by *luxI* and *luxR* is required to produce bistability (16). However, this study did not distinguish between cell-autonomous and population-level bistability, as it did not measure gene expression in single cells, and it did not cover the entire bistable region. The other study combined single-cell and population analysis to conclude that a single positive feedback by *luxR*, along with LuxR dimerization, is sufficient to produce cell-autonomous bistability (19). In this prior study, the role of *luxI*-mediated feedback alone was not evaluated. Responses were measured to fixed extracellular AHL rather than fixed cell densities, thereby masking *luxI*-based feedback and defining the properties of only one of two so-called nullclines of the system.

Many other studies have conducted QS gene expression measurements under dynamic growth conditions in standard batch cultures, which are generally easier to operate than steady-state cultures (25). Some studies show rapid, unimodal gene induction consistent with population-level bistability (26, 27), while most others show graded, heterogeneous, and in part bimodal induction patterns consistent with cell-autonomous bistability (28–34).

To better understand the steady-state QS response in a native context, we recently analyzed the *las* system of *P. aeruginosa* by mathematical modeling (13). We developed a deterministic mathematical model that contained the *las* system components described above, parametrized the model with dynamic, population-level gene expression data, and simulated the ensuing steady-state response. We found that the *las* QS system is bistable at the population level, with distinct on and off states, and that it exhibits hysteresis, with the on/off transition occurring at a lower cell density than the off/on transition.

In this study, we set out to experimentally test the prediction that the *las* QS system of *P. aeruginosa* constitutes a canonical, bistable population switch. We employed a recursive dilution scheme that enables growth under quasi-steady-state conditions. QS gene expression was measured with a green fluorescent protein (GFP) reporter at the population and single-cell levels. Our study demonstrates population-level bistability in a native QS system and shows that QS can indeed function as a collective gene expression switch, despite reports emphasizing cell-to-cell heterogeneity. This work has implications for our understanding of the functional capacity of QS in basic and applied contexts.

## RESULTS

### Gene expression reporter system

For most experiments, we chose the *lasI* synthase gene to measure the activity of the *las* QS system. We quantified *lasI* expression via a transcriptional reporter in which the *lasI* promoter with its LasR binding site is fused to the gene encoding GFP (P*lasI::gfp*). The reporter fusion is carried on a low-copy plasmid, and the *gfp* gene encodes a stable and fast-folding variant (35). This construct permits a sensitivity and induction range not achievable with single-copy reporters or with unstable GFP variants, particularly at the low cell densities required here (26, 33, 36). In terms of gene regulation, the contribution of plasmid-encoded LasR binding sites is considered to be minor, given that LasR binds over 30 target promoters in the *P. aeruginosa* genome (37, 38).

Stable GFP is fully compatible with our experimental approach and enables measurements of off-to-on but also on-to-off transitions. The levels of stable GFP in the cell are a function of biosynthesis and dilution by cell division, whereas proteolytic degradation is negligible (39). However, because steady-state cultivation is essentially time-independent, gene expression decreases can be appropriately captured by depleting GFP through multiple rounds of cell division until a new steady state is reached. At that point, GFP synthesis and dilution rates are again in balance.

Our plasmid-based system is also suitable for single-cell measurements. Its tight replication control results in a unimodal distribution across cells with gene expression patterns similar to chromosomal constructs (27, 33, 40). This property rules out the possibility that cell-to-cell heterogeneity, if observed, would be caused by differences in plasmid copy number. In addition, for single-cell applications, stable GFP is preferred over short half-life variants with complex degradation kinetics that can skew fluorescence intensity distributions across cell populations (39).

### Nonlinear *lasI* expression in carbon-limited batch cultures

**Fig 2:**
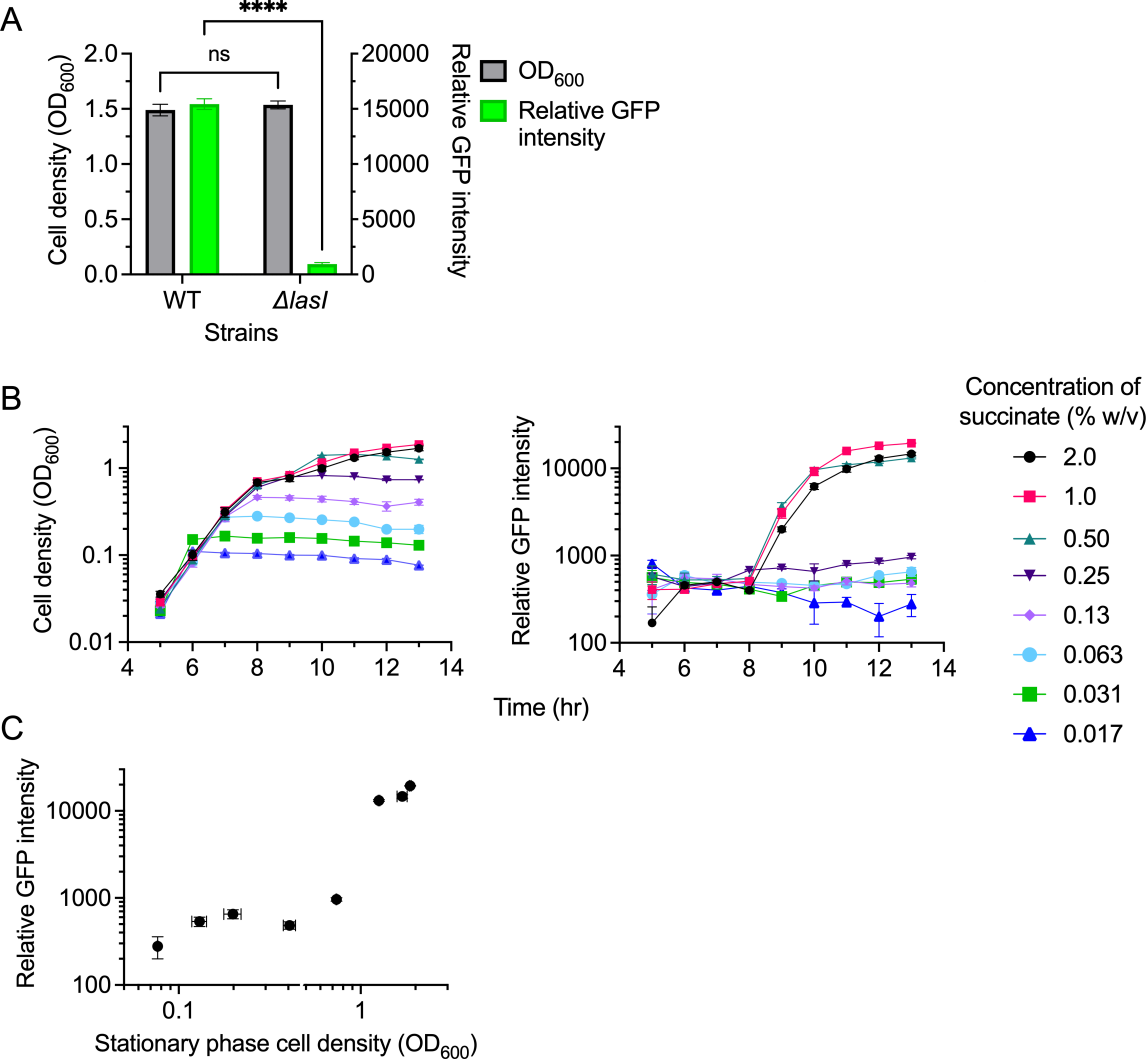
Growth and P*lasI::gfp* expression of *P. aeruginosa* in minimal medium batch culture with varying concentrations of succinate. Cells were grown in a 96-well plate format. (**A**) Stationary-phase cell density (OD_600_) and relative GFP intensity (GFP/OD) of the PAO1 WT and *ΔlasI* mutant grown in M9 medium with 1% succinate (wt/vol). ns, not significant and ****, *P* < 0.0001 according to *t*-test. (**B**) Cell density (left panel) and relative GFP intensity (right panel) of the WT grown at varying concentrations of succinate ranging from 0.017% to 2.0%. (**C**) Relative GFP intensity vs. stationary phase cell density (as measured in (B) after 13 h). *n* = 3, error bars indicate SD and are too small to be seen in some instances.

As a first step, we measured P*lasI::gfp* expression in batch culture populations grown to different saturating cell densities. These are not steady-state conditions, but they allowed us an initial assessment of the QS response as a function of stationary cell density, akin to a previous study (28). We achieved different carrying capacities by using a minimal medium (M9) that contained varying concentrations of succinate as the limiting carbon source. We initially compared relative GFP intensity in wild-type (WT) and Δ*lasI* mutant strains grown to high cell density at 1% succinate (Fig. 2A) to show that the *lasI* gene is significantly induced by LasR-3OC12-HSL under our growth conditions, consistent with previous data in rich medium (26, 27).

 We then measured GFP intensity of WT cultures during growth at different succinate concentrations, ranging from 0.017% to 2.0%, in twofold increments (Fig. 2B). Our particular interest was in the cell densities and P*lasI::gfp* levels achieved upon cessation of growth. We found that saturating cell density values increased linearly with succinate concentration (up to 1%, above which succinate was no longer the limiting nutrient), whereas P*lasI::gfp* levels were highly nonlinear (Fig. 2B; Fig. S1A). Succinate concentrations up to 0.25% resulted in expression levels near baseline, whereas succinate concentrations of 0.5% or higher resulted in high levels of induction (Fig. 2B, right panel). This nonlinearity is evident from a plot of relative GFP intensity vs. stationary phase cell density (Fig. 2C). A Hill-type sigmoidal function fit the data with a Hill coefficient of *n*_H_ = 6.1 showing that this is a highly cooperative, nonlinear response (Fig. S1B). For a gradual response type, we would expect a Hill coefficient approximately equal to 1. Taken together, these data suggest a nonlinear response of the *las* QS system to changes in cell density.

### Bistable population-level *lasI* expression at steady state

**Fig 3:**
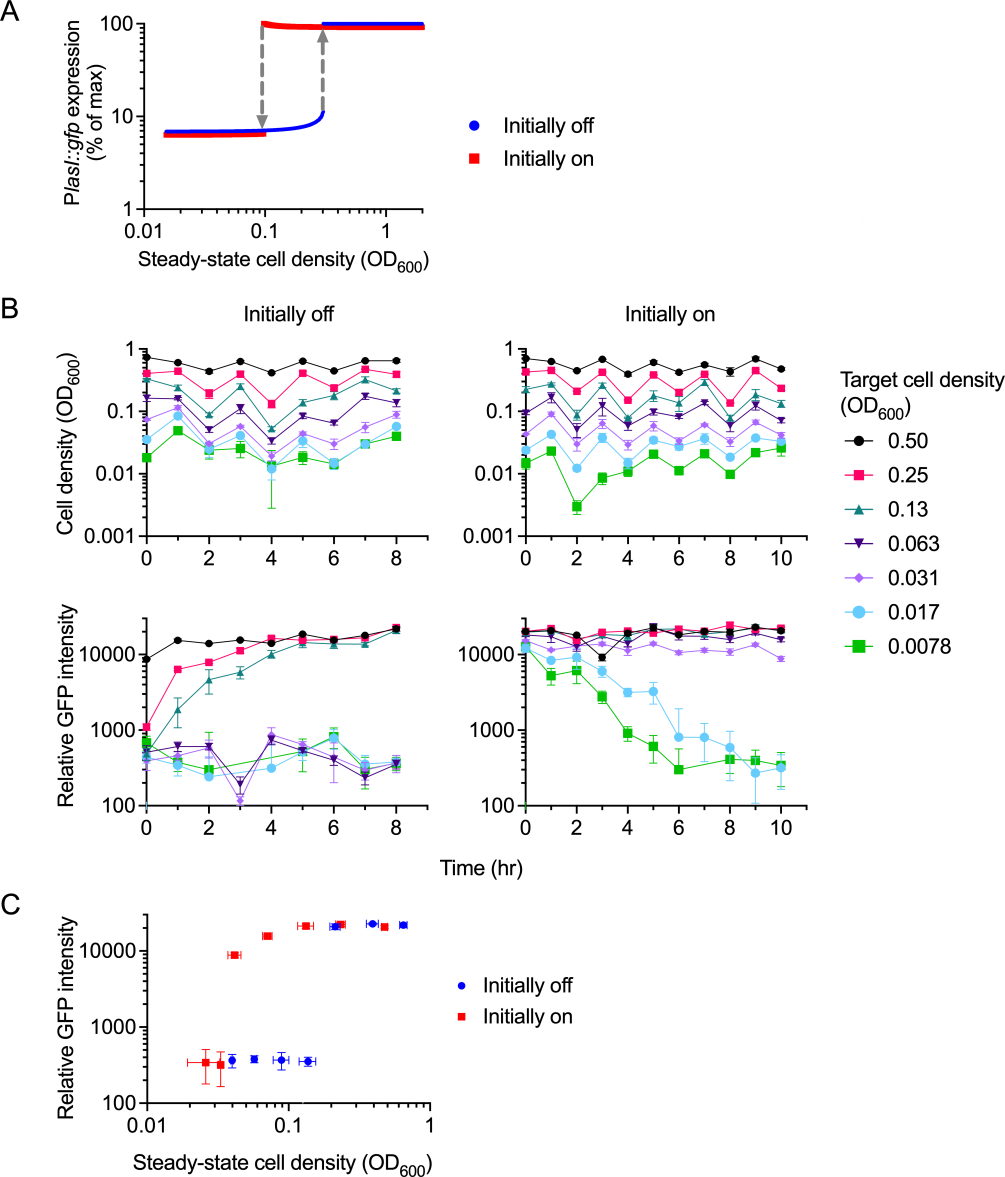
Steady-state *PlasI::gfp* expression. (**A**) Mathematical model parametrized with dynamic gene expression data from rich medium batch culture (13), showing bistability with on/off transitions at distinct cell densities according to the initial state (gray arrows). (**B**) Steady-state cultivation and gene expression of the PAO1 WT harboring the P*lasI::gfp* reporter. Cells were grown in 96-well plates containing M9 medium with 1% succinate. A range of cell densities was maintained by diluting cultures back to designated target densities every hour. Cultures were initiated either from low-density precultures (initially in the off-state, left panels) or from high-density precultures (initially in the on-state, right panels), as specified in Materials and Methods. Cell density was measured as OD_600_ (top panels), and gene expression was measured as relative GFP intensity (GFP/OD, bottom panels). Time 0 is defined as the first time that cultures exceeded, and were diluted back to, their target density. (**C**) Relative GFP intensity vs. cell density, as measured at the final time point. *n* = 3, with error bars indicating SD.

To directly test if the observed nonlinear population response is bistable, we conducted steady-state cultivation experiments. Our approach was guided by our recent mathematical modeling work suggesting that P*lasI::gfp* expression exhibits population-level bistability in response to steady-state cell density, with stable on and off states (13) (Fig. 3A; see Materials and Methods for a description of the model). Importantly, bistability endows the system with hysteresis. In the case of population-level bistability, this is evident as two distinct on/off transition points depending on the direction of the cell density change (low to high vs. high to low) (Fig. 1B, right panel). Accordingly, we designed our cultivation experiments such that they had the capacity to reveal hysteresis. We initiated steady-state cultures either from low density (P*lasI::gfp* expression low, herein referred to as “initially off”) or from high density (P*lasI::gfp* expression high, herein referred to as “initially on”).

To achieve steady-state conditions, we employed a periodic dilution batch cultivation scheme in microtiter plate format, modeled after a previous study (16). Cultures were maintained within a range of different optical densities by hourly dilution back to the original threshold density (OD_600_ of 0.0078–0.50, in twofold increments; Fig. 3B). Given the large number of samples, this approach was chosen over continuous-culture bioreactors as the more feasible and economical alternative. Cultures were grown in M9 medium containing a concentration of succinate that did not limit growth (1%), because in this case specific target densities were achieved by periodic dilution rather than by substrate limitation. To minimize biofilm formation, cultures were transferred to new wells every 2 h, contributing to the observed periodic fluctuation in cell density (Fig. 3B, top panels).

Using this process, we cultivated *P. aeruginosa* harboring PlasI::gfp for 8–10 h. Cultures were initiated either from off or on pre-cultures. While we were able to maintain cultures within distinct, non-overlapping cell density ranges in approx. 2-fold increments, we found that their relative GFP intensities approached either a low or a high expression level (Fig. 3B, bottom panels). This bifurcation was apparent in both cases, but the threshold cell density separating low and high expression levels differed significantly (Fig. 3C; Fig. S2). Cultures initiated from low density switched from off to on at an OD_600_ of approx. 0.2, and cultures initiated from high density switched from on to off at an OD_600_ of approx. 0.04. Our data also confirm that induced GFP-expressing cells were able to fully turn off within the experimental timeframe if the steady-state cell density was sufficiently low.

With respect to the kinetics of state-switching, cultures at intermediate target densities closest to the bistable region tend to take the longest time to switch and reach steady-state gene expression levels, regardless of the initial condition. This is consistent with theory, as these cultures experience the smallest cell density change that drives a state transition (13).

We found that the observed steady-state gene expression pattern with two distinct transition points is qualitatively very similar to that of our mathematical model (Fig. 3A), in support of population-level bistability. The on/off cell density thresholds are somewhat different between the model and experiment, which can be attributed to the fact that the model was parametrized based on experiments conducted with rich growth medium (13), whereas experiments in the current study were conducted with minimal medium. Taken together, our population-level data suggest that the *las* QS system functions as a bistable gene expression switch that exhibits hysteresis. We identified a sizable region of bistability, where populations can be either on or off at a given cell density, depending on their initial condition.

### Single-cell *lasI* expression at steady state

We aimed to compare population-level P*lasI::gfp* expression with single-cell P*lasI::gfp* expression. Theory predicts that single-cell responses producing population-level bistability should be unimodally distributed, with all cells switching between on and off states in a concerted fashion (20). We performed the above steady-state cultivation scheme with larger volumes suitable for single-cell measurements by flow cytometry. We focused on cell densities near the off/on and on/off transition points, guided by our population-level P*lasI::gfp* measurements.

**Fig 4:**
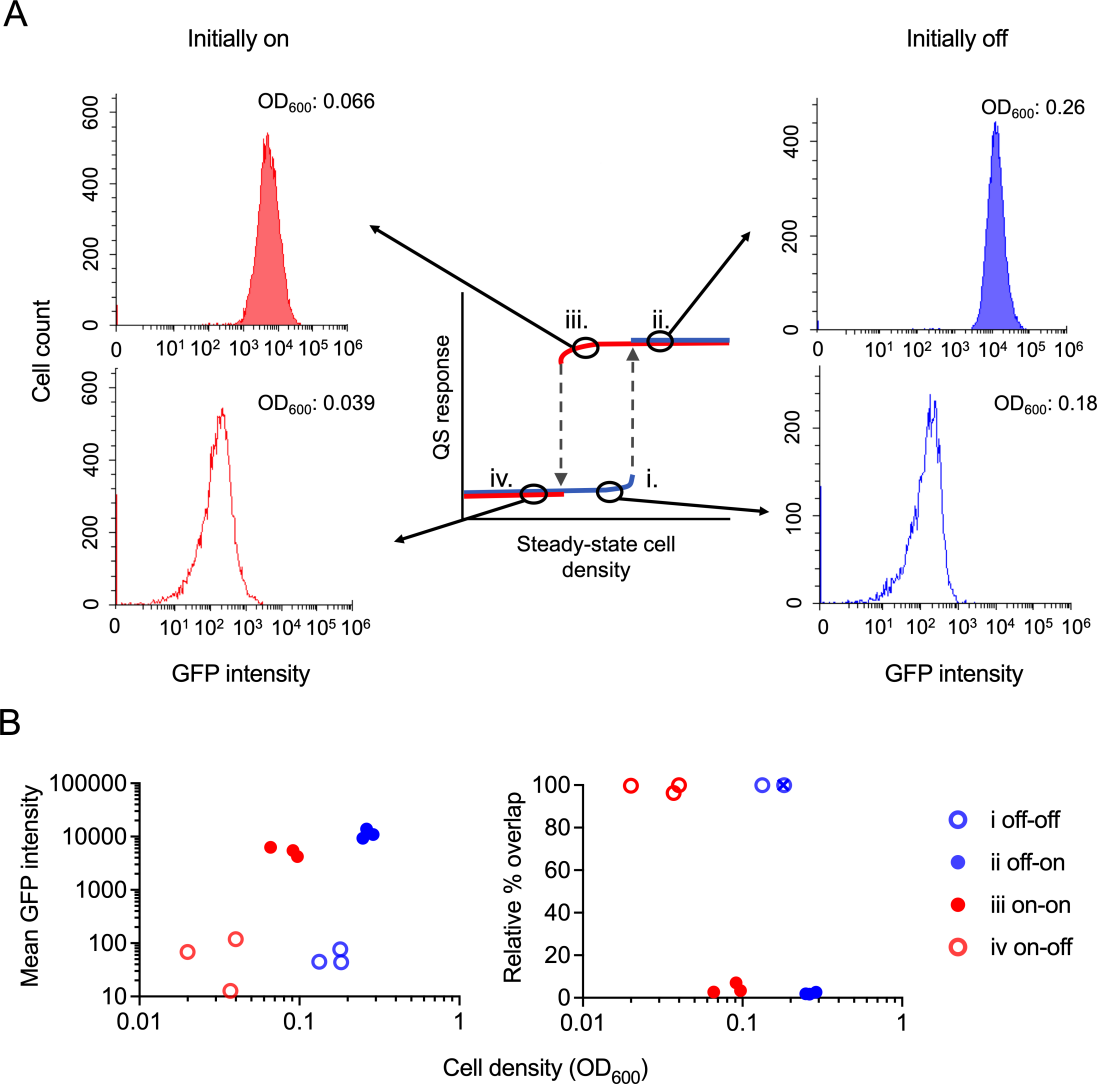
Single-cell analysis of QS activation states near transition thresholds by flow cytometry. PAO1 WT was grown at steady state in culture flasks containing M9 medium with 1% succinate. Samples were taken at the off (i) to on (ii) and on (iii) to off (iv) transition points. (**A**) Histograms of single-cell P*lasI::gfp* measurements from conditions i to iv plotted as cell count vs. GFP intensity. On-populations are solid, and off-populations are open (blue if initially off and red if initially on). The corresponding steady-state culture density (OD_600_) is shown in the upper right-hand corner of each histogram. (**B**) Mean GFP intensity of each replicate population (left panel) and relative percentage of cells overlapping with a designated off-state replicate population (right panel; designated sample with blue cross) plotted against steady-state OD_600_. *n* = 3.

We found that P*lasI::gfp* expression in individual cells showed a unimodal, homogeneous distribution of cells that were either all in the off-state or all in the on-state (Fig. 4). State-switching occurred within a narrow window of cell densities (OD_600_): from off to on (condition i to ii) between 0.18 and 0.26, and from on to off (condition iii to iv) between 0.066 and 0.039. The mean GFP intensities were significantly different between on and off states, in both directions (Fig. 4B, left panel, and Fig. S3, left panel), and the population overlap between on- and off-state samples was minimal to none (Fig. 4B, right panel and S3 right panel). Importantly, the attributes of unimodal distribution and distinct GFP intensities applied to the two samples within the bistable region (i, off, and iii, on) (Fig. 4A). These results support the conclusion that the bistability observed here is population-level bistability rather than cell-autonomous bistability. Heterogeneous gene expression stemming from cell-autonomous bistability would have produced a graded induction pattern at the population level (Fig. 1B, left panel) (20), which is not what we observed (Fig. 3).

### mRNA levels of *lasI* and other QS target genes at steady state

**Fig 5:**
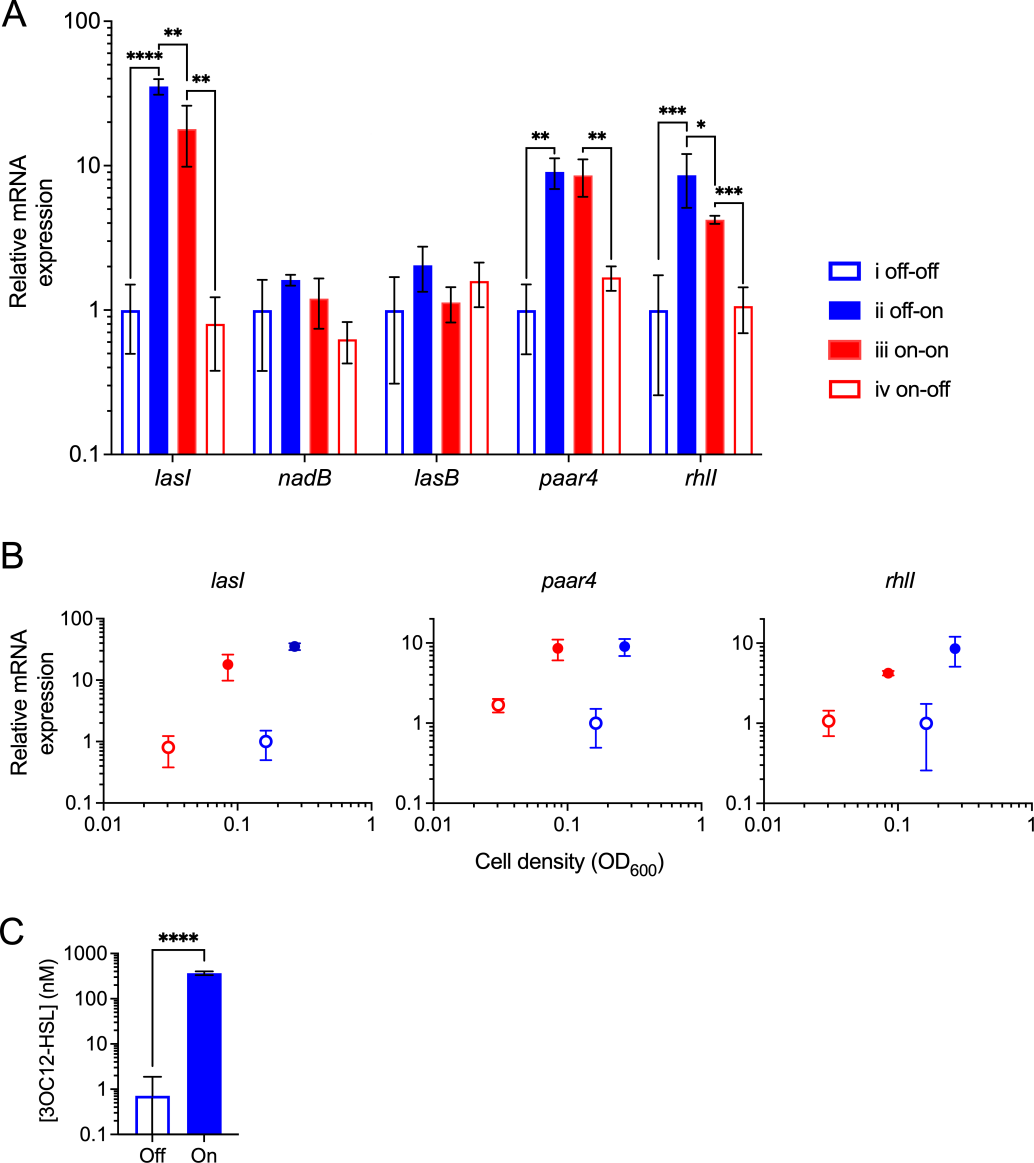
Transcript levels of QS-controlled genes at transition points. PAO1 WT was grown at steady state in culture flasks containing M9 medium with 1% succinate. (**A**) Relative mRNA quantities determined by qPCR for *lasI*, *lasB*, *paar4*, *rhlI*, and *nadB* (a QS-independent control gene). Statistical analysis by two-way ANOVA (no bracket, not significant; *, *P* < 0.05; **, *P* < 0.01; ***, *P* < 0.001; and ****, *P* < 0.0001). (**B**) Relative mRNA quantities for *lasI*, *paar4*, and *rhlI* plotted against steady-state cell density (OD_600_). For panels **A and B**, off-state cultures are open, and on-state cultures are solid (blue when initially off, and red when initially on). (**C**) Concentration of 3OC12-HSL determined by bioassay for cells in off or on state, taken only from cultures that were initially off. Statistical analysis by unpaired *t*-test (****, *P* < 0.0001). *n* = 3, with error bars indicating SD.

We again conducted large-volume steady-state cultivations to quantify transcript levels of *lasI* and other QS target genes by qPCR. Cultures were initiated with on- and off-state precultures, and measurements were taken around the off/on and on/off transition points as defined for *lasI* above. First, we intended to confirm that measurements conducted with our P*lasI* reporter fusion expressing stable GFP correlate with less stable mRNA levels. We found that steady-state *lasI* mRNA levels were significantly higher in the on state than in the off state (Fig. 5A), correlating with P*lasI::gfp* expression in microtiter plate cultures at the same cell densities (Fig. 3C), and with 3OC12-HSL concentrations quantified in large-volume cultures by bioassay (Fig. 5C). In support of bistability and hysteresis, sample pairs taken at similar cell densities within the bistable region (conditions i and iii) displayed distinct mRNA expression values, depending on the initial condition (Fig. 5A and B, *lasI* transcript).

 Second, we asked whether the expression pattern observed for *lasI* would extend to other genes in the LasR regulon. These genes included *paar4* encoding a type 6 secretion system tip protein, *lasB* encoding the exoprotease LasB, and *rhlI* encoding a second AHL synthase in the *rhl*-QS circuitry (9, 41, 42). We also included *nadB* as a control gene that is not affected by *las*-QS (37). The relative transcript levels of *paar4* and *rhlI* were significantly higher in the on-state than in the off-state, as expected, and sample pairs within the bistable region differed in their gene expression levels, as was observed for *lasI* (Fig. 5A and B, conditions i and iii). In contrast, *lasB* transcript levels did not significantly differ between on and of states. This gene is directly activated by LasR (12), but it is defined as a so-called late gene based on its induction pattern in batch culture (9). Late genes require an additional environmental trigger for induction, such as phosphorus or nitrogen starvation (43). These components are not limited in our steady-state cultures, where cells are grown at their maximum growth rates with sufficient amounts of nutrients. Taken together, these mRNA expression data confirm our GFP reporter fusion results and suggest that bistable behavior at the population level extends to other functions controlled by *las*-QS.

## DISCUSSION

In this study, we have experimentally described the *las-*QS response of the model organism *P. aeruginosa* as a population-level bistable switch. Our work proves theoretical predictions about the emergent behavior of QS systems. One such emergent behavior in biological systems is the long-term or steady-state response, which provides a framework for determining the effects of environmental changes, including population density changes, on physiology and ecology (24). In qualitative terms, QS systems are generally considered concerted gene expression switches (2, 3), and more specifically, mathematical modeling has predicted that some QS systems, including the *las* system in *P. aeruginosa*, function as a bistable switch with two stable steady states, on and off (13, 19, 44–46). The experimental testing of these important predictions has lagged behind, in part because specialized steady-state culture conditions are required that are challenging to conduct in large numbers with multiple samples. Consequently, bistability has never been experimentally tested in a native QS system.

To overcome these challenges, we implemented a recursive dilution cultivation approach to measure population-level QS gene expression in a microtiter plate format under quasi-steady-state conditions. In these experiments, we found evidence for a sharp transition between QS on and off states, indicative of bistability. Importantly, we also found evidence of hysteresis in the system, which can only exist if the system is bistable (15, 19, 47). This memory response lets cells taken from a previously induced state remain induced to far lower cell densities compared to cells that have not been induced before.

Mechanistically, bistability and hysteresis in QS emerge from two major network elements: positive feedback on synthase expression and positive cooperativity associated with receptor dimerization (15, 17). Both conditions are met in the *P. aeruginosa las* QS system (13). A second positive feedback on receptor gene expression is not necessary for bistability, but it has been shown to enhance hysteresis and hence, the robustness of the switch (13, 19). The exact mechanism of LasR dimerization remains to be elucidated. Genetic analysis suggested that AHL binding is a requirement for dimerization (48), but more recent modeling and gene expression work suggested that AHL signals bind to a pre-formed dimer (49). In principle, both mechanisms have the potential to generate positive cooperativity (50).

To further probe the underlying design principles of native QS, one could genetically manipulate the network. Guided by predictions from mathematical modeling and experiments in heterologous systems (15, 16, 19, 22), one could examine mutants that lack *lasI* or *lasR* feedback loops. One approach would be to engineer a *lasI* promoter that lacks a LasR binding site (27). Insights gained from these experiments would allow predictions about emergent properties from network components alone and enable the design of synthetic networks with desirable response types, ranging from graded to switch-like.

Evidently, the population-level bistability we observed in this study emerges from bistability at the cellular level, and the role of extracellular signaling is to synchronize and homogenize cellular responses. This conclusion is supported by flow cytometry data from this study (Fig. 4) and from our previous work (26) showing unimodal distributions of cells in the on and off states. Hence, the circuit is tuned such that induction is largely triggered by the extracellular accumulation of signal. A change in network parameters such as the signal induction threshold could shift the response toward cell-autonomous bistability resulting from intracellular signal feedback (20). We have recently shown that antiactivator proteins likely have a role in this process (26). They prevent self-sensing by sequestering LasR and increasing the induction threshold.

The concerted switching of a unimodal population of cells under steady-state conditions contrasts with the more heterogeneous responses during batch cultivation reported by some studies (28–34). The latter is a highly dynamic environment in which noise in basal gene expression can lead to differences in the timing of induction in an exponentially growing population of cells (44). Steady-state cultivation does not capture this dynamic effect, as it is concerned with the long-term behavior of the system at different cell densities. Our flow cytometry data, however, do not suggest that steady-state gene expression is virtually identical in all cells (Fig. 4A). While the large majority of cells is clustered tightly around a mean, small proportions of cells on opposing histogram edges show expression differences of up to 100-fold.

When comparing the induction thresholds between steady-state and batch cultures, we found that steady-state cultures induce at a far lower cell density. This result is remarkable, given that our knowledge about QS is largely based on batch culture data, but it is not surprising if one considers the different culture “histories” in each case prior to induction. In batch cultures, population size increases exponentially from initially very low starting densities, such that QS signal accumulation lags behind growth. In steady-state cultures, population size remains constant, such that QS signal concentrations quickly reach a level that is proportional to cell density.

We found evidence of bistability and hysteresis for the central *las*-QS system gene, *lasI*, as well as two other QS target genes, *paar4* and *rhlI*. There is evidence that both target genes, like *lasI*, are directly activated by LasR-3OC12-HSL (9, 12, 51–53), such that the QS response is less likely to be masked by other regulatory responses. The bistable regulation of *rhlI* further indicates that the RhlR/RhlI system, and at least some target genes under its control, might also exhibit bistability.

* *What might be the potential benefits of the bistable control of QS target genes? Previous modeling work by Heilmann et al. suggested that the shapes of the cost-benefit curves for the production of QS-controlled public goods determine the optimal form of regulation (22). They theorized that public goods with a sigmoidal, cooperative benefit function (in which benefit initially accelerates with increasing concentration before it saturates) should exhibit bistable expression control, whereas public goods with a saturating, non-cooperative benefit function (in which benefit decelerates with increasing concentration) should exhibit graded expression control. It is intriguing to apply these considerations to *paar4*. This gene is part of an operon that encodes a contact-dependent T6SS effector delivery mechanism with the ability to kill other gram-negative bacteria (41). This activity, exerted by individual cells, would benefit the population as a whole and could consequently be considered a “public good.” It is plausible that T6SS activity has a sigmoidal benefit curve, if the benefit attained from harming other cells (through a combination of mechanisms such as initial encounter, nutrients attained from killed cells, or territorial expansion) accelerates with increasing density of T6SS-proficient cells.

One other *las*-controlled target gene, *lasB*, did not show significant differential expression under our steady-state cultivation conditions (Fig. 5A), which we attribute to its co-regulation by nutrient starvation pathways (43). Co-regulation could be direct or indirect via the *rhl* system that controls *lasB* expression along with the *las* system (9, 54). The *lasB* gene encodes a secreted endoprotease, a public good with a predicted sigmoidal benefit curve that would justify bistable regulation by QS under the appropriate conditions (41). Intriguingly, a recent study focused on *lasB* expression in *P. aeruginosa* reports a graded response to increasing cell density at the population level (28). This difference may have to do with the culture conditions used. The authors used batch cultures with different nutrient concentrations to achieve distinct maximum cell densities, similar to our initial approach (Fig. 2). It is possible that under these conditions, nutrient starvation rather than AHL signal accumulation was the actual gene expression trigger.

In dynamic real-world environments, the general ecological costs and benefits of bistable, hysteretic systems are related to the scale and duration of the stimulus. Away from the bistable region, these systems buffer against small changes but drive state transitions in response to larger, more sustained changes (55). This property can protect QS populations from unfavorable transitions in the presence of noise (such as fluctuations in cell density or signal concentration) (23). It can also ensure commitment to distinct physiological states (such as growth vs secretion), akin to the differentiation programs observed in the eukaryotic cell cycle or in microbial development (56, 57). On the other hand, the system cannot be finely tuned to gradual environmental changes and might become trapped in a non-optimal state (58).

In the context of pathogenesis and infection control, hysteresis implies that QS-controlled virulence genes, once induced, remain stably induced and require a substantial reduction in population size to turn them back off (16).

A phenotypic memory effect different from hysteresis has been described recently in the context of QS (59). It involves the carryover of QS proteins as the system transitions from on to off. Remaining LuxR or LuxI-type proteins in cells permit reactivation upon signal exposure. Because these proteins are diluted by cell division and degraded, reactivation is time-dependent. In contrast, hysteresis is an essentially time-independent property that preserves an existing steady state and resists change.

Taken together, our work provides insights into the functional capacity of AHL-QS in *P. aeruginosa*, with implications for bacterial physiology and pathogenesis, as well as systems and synthetic biology.

## MATERIALS AND METHODS

### Strains, plasmids, and growth conditions

All strains and plasmids used in this study are listed in Table 1. *P. aeruginosa* PAO1 (Iglewski lineage) was used as the WT strain. The *lasI* deletion mutant (*ΔlasI*) was derived from this parent strain (60). The low-copy-number promoter-probe plasmid pPROBE-AT was used as a reporter of QS gene expression (26, 61). It encodes the stable and fast-folding GFP variant *gfpmut1* (35) with its own ribosome binding site and start codon.

**TABLE 1:** List of strains and plasmids used in this study

Strain or plasmid	Property	Reference
*Pseudomonas aeruginosa*		
PAO1	Wild type (WT)	(60)
PAO1 Δ*lasI*	PAO1 derivative, Δ*lasI*, unmarked in-frame deletion	(27)
Plasmids		
pProbe-AT	Broad-host-range vector with a promoterless *gfp*, Cb^R^	(61)
pPSSL1	248 bp *lasI* promoter cloned into pProbe-AT	(26)

For routine maintenance, bacteria were grown in Lennox LB medium, either in liquid culture or on agar plates. For gene expression experiments, bacteria were grown in M9 minimal medium containing 1× M9 salts (62) (6.8 g/L disodium phosphate anhydrous, 3 g/L monopotassium phosphate, 0.5 g/L sodium chloride, and 1.0 g/L ammonium chloride), 1.0 mM magnesium sulfate, 0.1 mM calcium chloride, non-chelated trace elements, and 0.017–2.0% (wt/vol) disodium succinate as a preferred carbon source for *P. aeruginosa* (43). All cultures were incubated at 37°C, and liquid cultures were shaken at 250 rpm. The antibiotic carbenicillin (Cb) was added at a final concentration of 200 µg/mL for plasmid maintenance as appropriate.

### Batch and steady-state cultivation

For batch culture experiments, strains were first streaked onto LB plates and grown for 18–30 h. From these plates, precultures containing 4 mL of M9 medium (1% succinate) were inoculated to an OD_600_ of 0.00001. Precultures were grown for 10 h with shaking to an OD_600_ of 0.1–0.2. This low-density preculturing scheme ensured that cellular GFP was reduced to background levels (26). A 96-well black bottom microtiter plate (Greiner Bio-One) containing 200 µL of M9 medium at a range of succinate concentrations (from 0.0156% to 2.0% in twofold increments) was inoculated to a starting OD_600_ of 0.001, in triplicate. Microtiter plate cultures were grown, with lid, in a shaker incubator. OD_600_ and bulk GFP intensity measurements (λ_ex_ = 480 nm and λ_em_ = 535 nm) were taken every hour in a Tecan Infinite M200 multifunction plate reader. Relative fluorescence intensity was calculated by dividing the total fluorescence by the corresponding OD_600_. All values were corrected for background from a promoterless GFP control.

Steady-state cultivation experiments were based on a recursive dilution scheme described by Haseltine and Arnold (16). Precultures were grown in 4 mL of M9 medium with 1% succinate to either a low cell density (off state, OD_600_ of 0.1–0.2) or a high cell density (on state, OD_600_ of 1.5–2.0). From these precultures, 96-well black-bottom microtiter plates containing 200 µL of M9 medium (1% succinate) were inoculated at a range of starting cell densities, in twofold increments. For the samples initiated with off-state precultures, the initial OD_600_ values were from 0.1 to 0.0016. For the samples initiated with on-state precultures, the initial OD_600_ values were from 0.5 to 0.0078. Microtiter plate cultures were incubated, and OD_600_ and GFP intensity were measured every hour as described for the batch culture samples above. As culture densities crossed their assigned OD_600_ threshold value, they were diluted back to that value by withdrawing a specific culture volume and replacing it with the same volume of fresh medium. The assigned OD_600_ values were from 0.5 to 0.0078, in twofold increments, such that cultures inoculated from off-state precultures crossed this threshold after several hours of growth in batch mode, whereas cultures inoculated from on-state precultures crossed this threshold after the first hour. Time 0 was defined as the first time that the cultures were diluted after they had crossed their target OD_600_ thresholds. This process was repeated every hour, for up to 10 h, with transfer of cultures to new wells every 2 h.

The same recursive dilution scheme was applied in a large-volume format for mRNA, AHL, and single-cell gene expression analysis at the transition thresholds from off to on (i–ii) and on to off (iii–iv) (Fig. 4A). Experimental cultures were initiated from off-state or on-state precultures and were grown in 250 mL baffled flasks with 40 mL of M9 medium (1% succinate). Cultures were harvested after at least 6 h of recursive dilutions, when steady state was reached.

### Measurement of single-cell fluorescence by flow cytometry

Samples from large-volume cultures were concentrated either by vacuum filtration (for lower-density samples) or by centrifugation (for higher-density samples). For vacuum filtration, Vivaspin 500 filters with a 0.2 µM polyether sulfone membrane (Sartorius) were connected to a vacuum pump. Concentrated cells were then immediately fixed using 2.5% (wt/vol) paraformaldehyde in phosphate-buffered saline (PBS) using a previously established protocol (26, 27). Flow cytometry samples were diluted to an OD_600_ of 0.01 in PBS in a white flat bottom 96-well plate.

Cell fluorescence was quantified using a Beckman Coulter CytoFLEX S flow cytometer. Emission filters were set to FITC 525/40 nm for GFP. A manual threshold of 8,000 was set in the forward scatter height channel, and 20,000 instances were recorded for each sample. Data analysis was done in the Cytoexpert and Flow Jo software. Cells were selected by gating using scatter plots of FSC area vs. SSC area. The fluorescence data are presented in histogram format by plotting cell count vs. GFP intensity. The percentage of cells in each population overlapping with a designated off-state sample was calculated using the Overton cumulative histogram subtraction method in Flow Jo (63).

### AHL measurements

For AHL analysis, 5 mL samples from large-volume cultures in the on and off states were extracted twice with acidified ethyl acetate (13). Extracts were concentrated by evaporation to full dryness and then resuspended in fresh ethyl acetate. The 3OC12-HSL concentration of these samples was determined by an established *E. coli* bioassay containing *lasR* and a *lasB′-lacZ* reporter (11, 13, 64). β-Galactosidase activity of bioassay cultures was measured using the Galacto Light Plus Reporter Gene Assay (Thermo Fisher). 3OC12-HSL concentrations in the experimental samples were determined by comparison with a standard curve generated with synthetic 3OC12-HSL (Sigma-Aldrich).

### mRNA quantification using real-time PCR

Culture conditions for mRNA extraction were as described above. Approximately 5 × 10^8^ cells were harvested from each large-volume culture in the on-state and the off-state. RNA isolation, DNase treatment, cDNA synthesis, and real-time PCR were performed in principle as described previously (43, 65). Briefly, RNA was extracted using the QIAGEN RNA Protect Bacteria Reagent and RNeasy Mini Kit according to specifications for gram-negative bacteria. RNA purity was quantified using a 2100 Bioanalyzer Instrument. cDNA was synthesized from the extracted RNA and purified as described previously (43, 65).

Real-time PCR was performed using an ABI Prism 7500 Fast system with the SDS v2.3 software. The following cycling parameters were used: 10 min at 95°C followed by 40 cycles of 15 s at 95°C and 1 min at 60°C. Each PCR mixture contained 1× SYBR Green PCR Master mix (Applied Biosystems), 0.3 pM of each primer, and 1 ng cDNA. Relative transcript levels were determined by using the standard curve method, using purified DNA from *P. aeruginosa* PAO1 at 1–0.0001 ng (in 10-fold increments) in each reaction. Primers for each gene of interest are provided in Table S1.

### Mathematical modeling

Modeling data were taken from our recent work (13), in which we built and analyzed a deterministic mathematical model of the *P. aeruginosa las* QS system. The model consists of a system of ordinary differential equations, with intracellular and extracellular AHL, LasI synthase, LasR receptor, RsaL repressor, and cell density as state variables. The model was parameterized by fitting to dynamic, population-level AHL and GFP-reporter fusion data obtained from LB batch cultures. Steady-state expression of P*lasI::gfp* at constant cell densities was modeled as described (13). Cell densities given as CFU/mL in the model were converted to OD_600_ values in Fig. 3A by OD_600_ = 1.527 × 10^−9^ CFU/mL, as determined previously (13).

### Statistical analysis

Statistical analysis of experimental data was performed using GraphPad Prism (version 10.2.3 for Windows). The specific statistical test used is described in the respective figure legend. A *t*-test was performed as a two-tailed, unpaired Student’s *t*-test. One-way and two-way ANOVA tests were used, in some cases paired with Sidak’s multiple comparison analysis, comparing individual samples as indicated. The significance level α was 0.05.

## SUPPLEMENTAL MATERIAL

Supplemental figures and tables mbio.01713-25-s0001.pdf Figures S1 to S3 and Table S1.

ASM does not own the copyrights to Supplemental Material that may be linked to, or accessed through, an article. The authors have granted ASM a non-exclusive, world-wide license to publish the Supplemental Material files. Please contact the corresponding author directly for reuse.
