# Supplementary material for: Population-level bistability in Pseudomonas aeruginosa quorum sensing
Source: mBio. 2025 Sep 10;16(10):e01713-25. doi: 10.1128/mbio.01713-25 (PMC12505975; doi:10.1128/mbio.01713-25)
Supplement: Supplemental figures and tables — Figures S1 to S3 and Table S1. [file mbio.01713-25-s0001.pdf]

Supplemental Material, Pettit Estell and Schuster *mBio* 2025

Figures S1-S3

Table S1

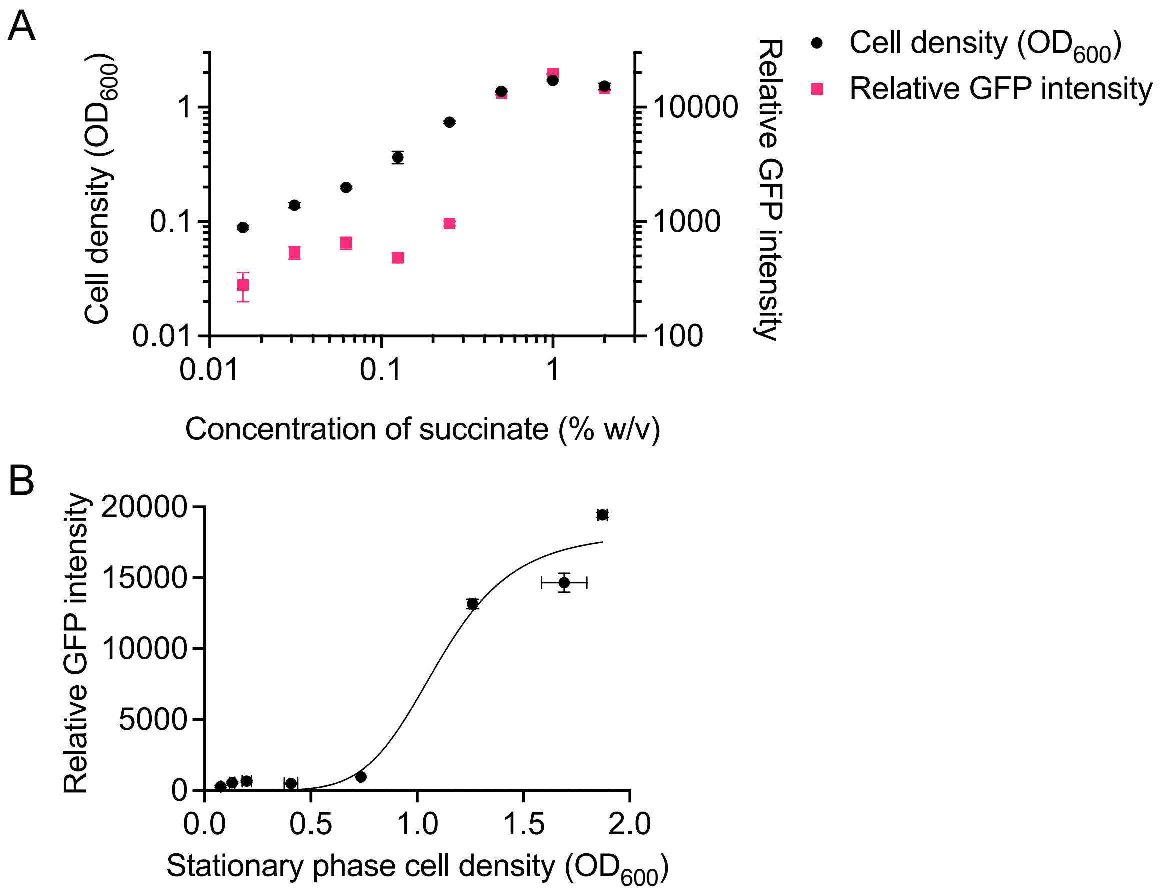

Figure S1. Non-linearity of P*lasI::gfp* expression in batch culture. A. Average final cell density (OD_{600}, black circles) and relative GFP intensity (GFP/OD_{600}, pink squares) as a function of succinate concentration (% w/v), taken from the data in Fig. 2B. **B.** Relative GFP intensity as a function of stationary phase cell density (OD_{600}), fit to a Hill-type sigmoidal function with a Hill coefficient of 6.14 ($r^2 = 0.98$). n=3, error bars indicate SD.

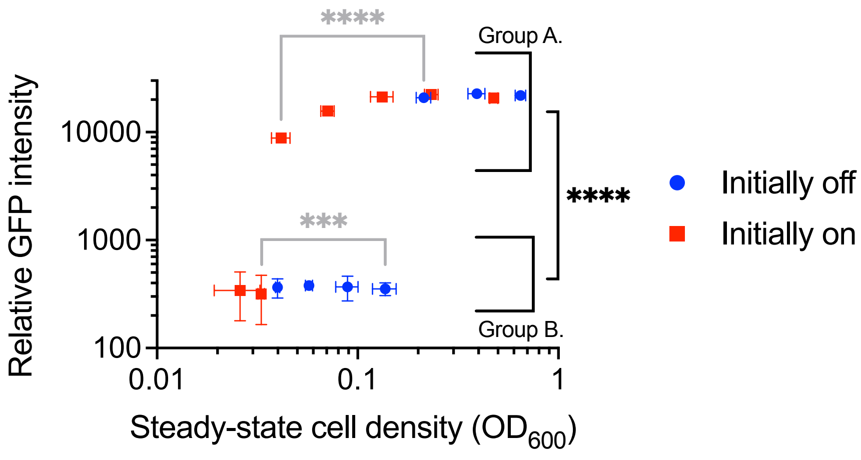

Figure S2. Statistical analysis of steady-state P*lasI::gfp* expression. Relative GFP intensity (GFP/OD_{600}) is plotted vs. steady-state cell density (OD_{600}; data from Fig. 3c). Two types of comparisons were made, as indicated by the respective brackets. First, samples of relatively high GFP intensity considered to be in the on-state (group A) were compared to samples of relatively low GFP intensity considered to be in the off-state (group B), using two-way ANOVA. There was a significant difference between the two groups (black vertical bracket, ****, $P < 0.0001$). Second, cell densities near the on/off transition were compared to those near the off/on transition using a t-test (grey brackets pointing to specific data points). There was a significant difference between each on-state transition density pair, and between each off-state transition density pair (***, $P < 0.001$; ****, $P < 0.0001$). n=3, error bars indicate SD.

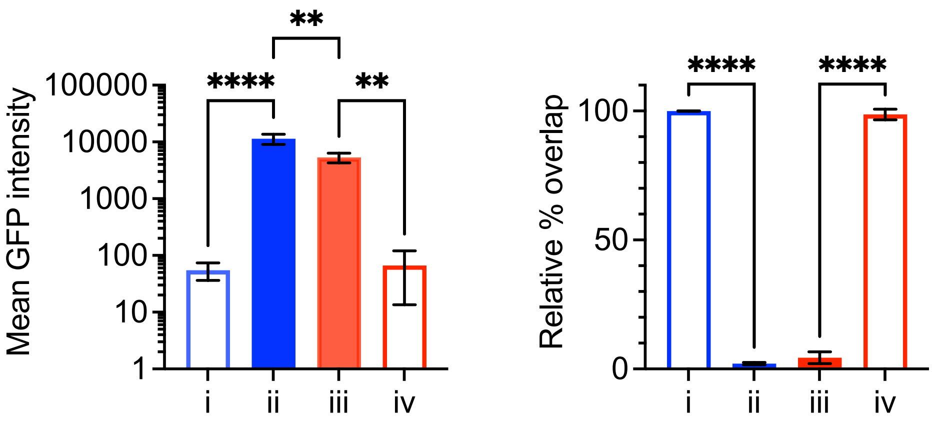

Figure S3. Statistical analysis of single-cell flow cytometry data. Mean GFP intensity (left panel) and percent histogram overlap (right panel) are shown for each growth condition, i to iv. Off-state cultures are open, and on-state cultures are solid (blue when initially off and red when initially on). All samples were compared to each other by one-way ANOVA (no bracket, not significant; **, $P < 0.01$; ***, $P < 0.001$; ****, $P < 0.0001$). n=3, error bars indicate SD.

Table S1. List of primers used for qPCR.

Construct or gene	Primer name (forward and reverse)	Primer sequence (5'→3')
lasI	lasI F qPCR_B050923	GGCGCGAAGAGTTCGATAAA
	lasI R qPCR_B050923	CCATCTCGTCGATGACACTAAC
rhlI	rhlI F qPCR_B050923	GCAGCTGGCGATGAAGATA
	rhlI R qPCR_B050923	GCCGTTGCGAACGAAATAG
lasB	lasB F qPCR_B050923	CAACCAGAAGATCGGCAAGTA
	lasB R qPCR_B050923	GTTCATGTCGACGGTGATGA
paar4	paar F qPCR_B050923	GACGTCTTCTTCGACCATCTG
	paar R qPCR_B052223	GGCTTGCCGTTGATCAGTA
nadB	nadB_F_081823bpe	GAACATCGAGTTGCTGTCGC
	nadB_R_081823bpe	ACGGAAGGTATCGACTTCGC
